# Antioxidant Activity, Antiproliferative Activity, Antiviral Activity, NO Production Inhibition, and Chemical Composition of Essential Oils and Crude Extracts of Leaves, Flower Buds, and Stems of *Tetradenia riparia*

**DOI:** 10.3390/ph17070888

**Published:** 2024-07-04

**Authors:** Jéssica da Silva Sena, Selma Alves Rodrigues, Karina Sakumoto, Rodrigo Sadao Inumaro, Pamela González-Maldonado, Emilio Mendez-Scolari, Ranulfo Piau, Daniela Dib Gonçalves, Filipa Mandim, Josiana Vaz, José Eduardo Gonçalves, Pablo Hernan Sotelo, Juliana Silveira do Valle, Zilda Cristiani Gazim

**Affiliations:** 1 Graduate Program in Biotechnology Applied to Agriculture, Universidade Paranaense, Umuarama 87502-210, Brazil; jessica.sena@edu.unipar.br (J.d.S.S.); jsvalle@prof.unipar.br (J.S.d.V.); 2 Graduate Program in Animal Science with Emphasis on Bioactive Products, Universidade Paranaense, Umuarama 87502-210, Brazil; selma.rod@edu.unipar.br (S.A.R.); danieladib@prof.unipar.br (D.D.G.); 3 Graduate Program in Medicinal and Phytotherapeutic Plants in Primary Care, Universidade Paranaense, Umuarama 87502-210, Brazil; karina.sakumoto@edu.unipar.br; 4 Graduate Program in Clean Technologies, UniCesumar, Maringá 87050-390, Brazil; rodrigo_inumaro@hotmail.com (R.S.I.); jose.goncalves@unicesumar.edu.br (J.E.G.); 5 Biotechnology Department, Facultad de Ciencias Químicas, Universidad Nacional de Asunción, San Lorenzo 111421, Paraguay; pame.54.pg12@gmail.com (P.G.-M.); emendez.scolari@gmail.com (E.M.-S.); phsotelo@qui.una.py (P.H.S.); 6 Centro de Investigação de Montanha (CIMO), Instituto Politécnico de Bragança, Campus de Santa Apolónia, 5300-253 Bragança, Portugal; filipamandim@ipb.pt (F.M.); josiana@ipb.pt (J.V.); 7 Laboratório Associado para a Sustentabilidade e Tecnologia em Regiões de Montanha (SusTEC), Instituto Politécnico de Bragança, Campus de Santa Apolónia, 5300-253 Bragança, Portugal; 8 Cesumar Institute of Science, Technology and Innovation, UniCesumar, Maringá 87050-390, Brazil

**Keywords:** Brazilian myrrh, monoterpene hydrocarbons, oxygenated sesquiterpenes, 14-hidroxy-9-epi-caryophyllene, diterpenol, rosmariniquinone, antiproliferative, cellular antioxidant, HSV-1

## Abstract

The chemical composition of extracts (CEs) and essential oils (EOs) from *Tetradenia riparia* leaves, flower buds, and stems was analyzed. Antiproliferative activity against tumor cell lines, NO production inhibition, and antioxidant and antiviral activities were assessed. The CEs contained flavonoids, phenolic acids, coumarins, and saturated fatty acids. The EOs included monoterpenes, oxygenated sesquiterpenes, and diterpenes. NO production inhibition ranged from 76 to 247 µg mL^−1^, and antiproliferative activity exhibited GI_50_ between 20 and >204 µg mL^−1^, with low cytotoxicity (SI: 1.08 to 4.75). Reactive oxygen species inhibition ranged from 45 to 82%. Antioxidant activity varied when determined by the 2,2-diphenyl-1-picrylhydrazyl radical scavenging assay (IC_50_: 0.51 to 8.47 mg mL^−1^) and ferric reducing antioxidant power (0.35 to 0.81 µM ferrous sulfate per mg). The reduction in β-carotene–linoleic acid co-oxidation varied between 76.13 and 102.25%. The total phenolic content of CEs and EOs was 10.70 to 111.68 µg gallic acid mg^−1^. Antiviral activity against herpes simplex virus type 1 (HSV-1) showed an EC_50_ between 9.64 and 24.55 µg mL^−1^ and an SI between 8.67 and 15.04. Leaf EOs exhibited an EC_50_ of 9.64 µg mL^−1^ and an SI of 15.04. Our study unveils the diverse chemical composition and multifaceted pharmacological properties of *T. riparia*, demonstrating its potential as a valuable source of bioactive compounds for therapeutic applications.

## 1. Introduction

The Lamiaceae family comprises various plants, most of which have uses in biology and medicine. The family encompasses 224 genera and is found worldwide, with over 5600 species [1].

Species belonging to this family are rich in essential oils, which hold great value in natural medicine, pharmacology, cosmetology, and aromatherapy. Essential oils are primarily located in the leaves but can also be found in flowers, buds, fruits, seeds, bark, wood, and roots [2]. Species in the Lamiaceae family are known for their medicinal and aromatic properties. One genus in this family is *Tetradenia*, which comprises 20 species [3], usually fragrant shrubs that grow up to 1–3 m tall. They are dioecious and soft and have many branches [4]. *Tetradenia riparia* is commonly known as *falsa-mirra*, *mirra-brasileira*, *incenso*, *lavândula*, *limonete*, or *pluma-de-névoa*. It has been introduced as an exotic ornamental plant in Brazil. Owing to its pleasant and intense aroma, it is cultivated in vegetable gardens, residential gardens, and parks [5].

Various authors have conducted chemical investigations on *T. riparia*. Weaver et al. [6] investigated the essential oil of *T. riparia* grown in Africa and discovered a complex terpenoid structure. A diterpene-type abietane, 9β,13β-epoxy-7-abietene, was isolated from the essential oil of *T. riparia* leaves grown in South America [7]. In the 1970s, Zelnik et al. [5] isolated ibozole and 7α-hydroxyroileanone from leaf extracts. Van Puyvelde et al. [8] isolated 1′,2′-dideacetylboronolide, and the diterpene diol 8(14),15-sandaracopimaradiene-7α,18-diol from leaf extract [9], showing antimicrobial activity. Davies-Coleman and Rivett [10] obtained leaf extracts and isolated 5,6-dehydro-α-pyrone (muravumbolide). The α-pirone tetradenolide was previously isolated from *T. riparia* [11]. The chemical structures and pharmacological information of these compounds are described in an extensive review carried out by our research group [4].

The essential oil gives this species an intense aroma in leaves, flower buds, and stems [4]. Recent studies have explored the chemical and biological properties of essential oils extracted from floral buds and stems. Zardeto et al. [12] were the first to report the action of essential oils from leaves, floral buds, and stems in controlling *Rhipicephalus sanguineus* larvae and their larvicidal activity against *Aedes aegypti* [13]. The leaves, floral buds, and stems were also investigated by Scanavacca et al. [14], who found antifungal and anti-mycotoxigenic activities, and by Cella et al. [15], who highlighted the acaricidal and larvicidal activities against *Rhipicephalus (Boophilus) microplus*.

The growing demand for products with fewer health risks has led to the search for natural and organic ingredients in processed foods and beverages, personal hygiene products, and beauty products [16,17]. This trend has boosted the industrial essential oils market, valued at over USD 7.51 billion worldwide in 2018 and expected to grow by 9% by 2026 [18].

Numerous studies have shown that various essential oils (EOs) possess antioxidant properties [7,19,20,21,22,23,24]. These investigations are a fast-growing area of research [25], as synthetic antioxidants have been linked to health issues such as allergies, cardiovascular and gastrointestinal diseases, and even cancer [26]. Natural antioxidants such as essential oils are becoming increasingly important.

Research into the biological activities of plants, such as their chemopreventive, antineoplastic, and antiviral actions, is of utmost importance in contemporary pharmacology [27,28,29]. Notably, around 25% of traditional antineoplastic drugs originate from plants, and another 25% are altered forms of phytopharmaceutical compounds, underscoring the potential of plant-based compounds as therapeutic agents [30]. Therefore, the diversity of bioactive compounds produced by plants, including flavonoids, alkaloids, terpenoids, and polyphenols, offers a rich reservoir for exploring novel biological activities.

This study evaluated the antioxidant, antiproliferative, antiviral, and NO production inhibitory activities of essential oils and crude extracts from the leaves, flower buds, and stems of *T. riparia*.

## 2. Results

Three anthocyanins and three flavonoids were identified in the crude extracts of leaves, flower buds, and stems. The leaves contained seven phenolic acids, the flower buds contained six, and the stems contained four. The presence of tannins was only evident in the leaves. Terpenes comprised the predominant class, with 16 being identified in the leaves, 14 in the flower buds, and 12 in the stems (Table 1).

Table 2 presents the chemical components found in the essential oils derived from the leaves, flower buds, and twigs of *T. riparia*. Oxygenated sesquiterpenes were the major class of EOs in leaves (35.0%), floral buds (41.2%), and stems (50.5%). In this class, α-cadinol and 14-hydroxy-9-epi-caryophyllene stood out as the predominant compounds in the leaves (12.2 and 8.6%, respectively), flower buds (10.7 and 14.2%, respectively), and stems (7.4 and 5.1%, respectively). It is also noteworthy that 9β,13β-epoxy-7-abietene and 6,7-dehydroroyleanone, two oxygenated diterpenes, were present in the leaves (7.3 and 5.8%, respectively), flower buds (8.8 and 7.5%, respectively), and stems (4.3 and 7.5%, respectively). Fenchone, an oxygenated monoterpene, was identified in the leaves (11.6%) and flower buds (4.7%).

Table 3, Table 4 and Table 5 show the antioxidant activities of the EOs and crude extracts. Table 3 shows the inhibitory potential of the EOs and crude extracts on the co-oxidation of β-carotene–linoleic acid. The leaf crude extract had the highest inhibitory effect on oxidation at the tested concentrations (102.25 to 95.98%), followed by essential oil from flower buds (80.28 to 74.41%).

Table 4 shows the regeneration action of DPPH• free radicals, the ferric reducing antioxidant power (FRAP) of essential oils and crude extracts, and the determination of total phenols (TPs) contained in the samples. For the DPPH assay, the stem crude extract showed a significant effect on the DPPH free radical (0.51 mg mL^−1^), followed by floral buds (0.91 mg mL^−1^), when compared with the quercetin control (0.01 mg mL^−1^). The samples did not show significant results in the FRAP assay compared with the Trolox control (9.17 µM ferrous sulfate mg^−1^ sample). Higher total phenol (TP) levels were observed for crude extracts, in particular the stem crude extract (111.68 µg GAE mg^−1^ of the sample).

Antioxidant activity was assessed in murine macrophage cell cultures (RAW 264.7), which showed 82% and 64% inhibition at the maximum concentration tested for crude leaf extract and essential oil, respectively, as shown in Table 5.

Essential oils and crude extracts of *T. riparia* also inhibited nitric oxide (NO) production in murine macrophage cells (RAW 246.7), as shown in Table 6. Higher inhibition was observed for essential oil from stems (76 µg mL^−1^), followed by leaf essential oil (95 µg mL^−1^).

The antiproliferative potential of the essential oils and crude extracts is shown in Table 7. Essential oils and crude extracts showed greater antiproliferative capacity against AGS (GI_50_ 20.00–67.00 µg mL^−1^) and CaCo-2 (GI_50_ 41.00–107.00 µg mL^−1^) cells. For the MCF-7 cell line, the crude extracts from the leaves and stems presented GI_50_ values of 59.00 and 72.00 µg mL^−1^, respectively, showing a greater antiproliferative action. The antiproliferative effect was also verified for the NCI-H460 cell line in stem EO and CE (GI_50_ 55.00 and 75.00 µg mL^−1^), and flower bud and leaf CEs (GI_50_ 74.00 and 77.00 µg mL^−1^).

**Figure 1:**
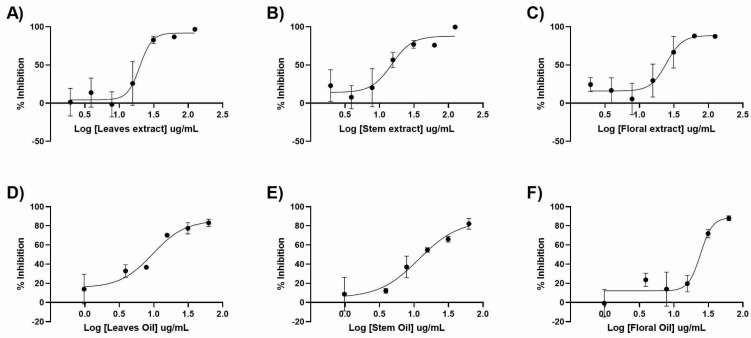
Antiviral activity of extract and essential oils of *T. riparia*. Vero cells were infected at MOI 1.5, followed by the addition of the corresponding extract or essential oil. The virus genome production in the supernatant was quantitated by using qPCR after 48 h. The percentage of inhibition was determined as the ratio between treated and untreated infected cells. (**A**) leaves extract, (**B**) stem extract, (**C**) flower extract, (**D**) leaves essential oil, (**E**) stem essential oil, and (**F**) flower essential oil. The results represent the means ± standard deviation of three separate assays.

Furthermore, *T. riparia* antiviral activity against HSV-1 was evaluated. For this purpose, serial dilutions of the extracts or EOs were added following virus adsorption, and the quantity of virus generated was evaluated by using qPCR. The findings, shown in Figure 1 and Table 8, reveal no significant differences between the EOs and the extracts. The leaf EO was the most active product, with an EC_50_ of 9.6 µg mL^−1^ and an SI of 15, while the flower bud extract was the least active product, with an EC_50_ of 24.55 µg mL^−1^ and an SI of 7.49.

## 3. Discussion

### 3.1. Antioxidant Activity

The biological potential of essential oils and crude extracts from various parts of the *T. riparia* plant was assessed in this study. The antioxidant potential of this species is strengthened by the presence of anthocyanins, phenolic acids, flavonoids, tannins, and terpenes in the crude extracts, which are discussed in this section.

The crude extract of the leaves showed the most significant inhibition of oxidation, reaching 102.25% in the β-carotene–linoleic acid co-oxidation system and 82% in cellular antioxidant activity. The high percentage of lipid peroxidation inhibition of the leaf crude extract can be attributed to the presence of procyanidins, which were exclusively identified in this extract.

Faria et al. [31] evaluated the effectiveness of five procyanidin fractions in preventing lipid peroxidation induced by 2,2′-azobis-2-methyl-propanimidamide dihydrochloride in a liposomal membrane system. They evaluated the antioxidant capacities of all fractions by observing the oxygen consumption and measuring the formation of conjugated dienes. The results showed that all tested fractions extended the oxidation induction time, protecting the membranes against peroxyl radicals. One likely explanation for this protective effect is that the phenolic substances found in the extracts capture peroxyl radicals, preventing the start of lipid peroxidation.

The presence of protocatechuic acid identified only in the CE of the leaves (Table 1) may explain this result. Li et al. [32] describe that this phenolic acid was more effective than the positive control, Trolox, in two different methods: DPPH (IC_50_ = 1.88 μg mL^−1^ for protocatechuic acid and 5.28 μg mL^−1^ for Trolox) and ABTS (IC_50_ = 0.89 μg mL^−1^ and 2.08 μg mL^−1^, respectively).

The stem crude extract exhibited the most significant regenerative impact on the DPPH radical, with an IC_50_ value of 0.51 ± 0.03 mg mL^−1^, and it also had the highest level of total phenols at 111.68 µg of gallic acid per mg of the sample (Table 4). A probable explanation is that eugenol was found only in this extract. This justification is corroborated by Dawidowicz and Olszowya [33], who reported the antioxidant activity of eugenol with potential against the radicals DPPH (IC_50_ = 0.1967 mg mL^−1^) and ABTS (IC_50_ = 0.1492 mg mL^−1^).

Fernandez et al. [34] performed chromatographic fractionation of the crude extract of *T. riparia* leaves. Two fractions stood out in terms of antioxidant potential, where the first was composed of the flavonoids astragalin and luteolin and an alpha-pyrone boronolide with 181.67 µg gallic acid mg^−1^ of the sample and the second was composed of diterpenol or ibozole with 119.85 µg gallic acid mg^−1^ of the sample. These same fractions provided a regenerative effect of the DPPH radical with IC_50_ = 0.61 and 0.88 mg mL^−1^, respectively, by the DPPH method and provided 55.61 and 39.57% protection from oxidation by the β-carotene–linoleic acid system and 4.59 and 2.23 µM of ferrous sulfate per mg of sample by FRAP, respectively. A third fraction, containing another diterpene, abieta-7,9 (11)-dien-13-b-ol, showed 80.15% protection from oxidation by the β-carotene–linoleic acid system.

The terpenes found in the crude extracts of the leaves, flower buds, and stems support the antioxidant action, in particular the phenolic diterpenes carnosic acid, carnosol, rosmanol, and rosmarinidiphenol. The protective effects of these phenolic diterpenes against lipid peroxidation in soybean oil were evaluated by Richheimer et al. [35] The results indicated that carnosic acid provided greater protection than butylated hydroxytoluene (BHT), butylated hydroxyanisole (BHA), and tertiary butylhydroquinone (TBHQ).

*Tetradenia riparia* contains an essential oil rich in sesquiterpenes and oxygenated diterpenes (Table 2). This may also have corroborated the antioxidant potential of the EO extracted from the leaves, flower buds, and stems.

Two compounds were extracted from the essential oil of leaves of *T. riparia*: 9β,13β-epoxy-7-abiethene, and 6,7-dehydroroileanone [7]. The antioxidant potential of the essential oils and compounds was examined by using the DPPH method. The essential oil exhibited an IC_50_ value of 15.63 µg mL^−1^, while the 6,7-dehydroroileanone compound showed a value of 0.01 µg mL^−1^. Their protective effects were also assessed by using the β-carotene–linoleic acid system and the 2,2′-azinobis-(3-ethylbenzothiazoline-6-sulfonic acid) (ABTS) method. The results indicated 130.1% and 109.6% inhibition for the β-carotene–linoleic acid system and 1524 μM Trolox g^−1^ and 1024 μM Trolox g^−1^ for the ABTS method, respectively. The results indicate that 6,7-dehydroroileanone, a diterpene responsible for the orange color of the essential oil, has a high antioxidant potential.

The ability of extracts to protect cells against oxidative damage is assessed by cellular antioxidant activity (CAA). This approach was created to address the necessity of using a cell culture model to evaluate the potential in vivo antioxidant capabilities, which would be more biologically relevant compared with chemical antioxidant assays [36].

The in vitro assay conditions may differ from those in a living organism and do not account for how substances are absorbed or metabolized. Additionally, how antioxidants work extends beyond their ability to counteract free radicals to prevent disease and promote health. However, using animals and conducting studies on humans are costly and unsuitable for the initial evaluation of biomolecules, foods, and supplements. Therefore, it is necessary to use cell culture models to aid antioxidant research prior to conducting studies on animals and clinical trials involving humans [37].

### 3.2. NO Production Inhibition

Regarding the inhibition of nitric oxide (NO) production (Table 6), the EO of the stems showed the greatest inhibition (EC_50_ = 76 µg mL^−1^), followed by the leaf EO, with EC_50_ = 95 µg mL^−1^. The presence of the oxygenated sesquiterpene spathulenol (9.09% in *T. riparia* stem EO and 2.93% in leaf EO (Table 2)) may have contributed to this result, as Subedi et al. [38] suggested in their studies that spathulenol shows considerable activity against NO production induced by lipopolysaccharide (LPS) in RAW 264.7, suggesting the possible effectiveness of this substance in the treatment of neurodegenerative conditions.

The sesquiterpene spathulenol was observed in the n-hexane fraction obtained from the EtOH:H_2_O (70:30) extract of the aerial parts of *Oliveria decumbens* Vent. The fraction containing spathulenol was assessed for its ability to inhibit the production of nitric oxide induced by lipopolysaccharide in murine macrophages. The results indicated significant dose-dependent anti-inflammatory activity in LPS-stimulated cells [39].

β-Caryophyllene, one of the primary compounds found in leaf (5.7%) and stems (4.99%) EOs (Table 2), may also have acted to inhibit the production of nitric oxide. A previous study assessed the anti-inflammatory impact of this substance by using an animal model supported by fluorescence molecular tomography. They reported a noteworthy decrease (*p* < 0.01) in edema volume and lower fluorescent signal intensity than the control [40].

Hernandez-Leon et al. [41] showed that β-caryophyllene (3.16–10 mg kg^−1^) has a dose-dependent antinociceptive effect in animal models and inhibits nitric oxide production. Furthermore, the research study affirmed that the pain-relieving properties of β-caryophyllene are facilitated by opioid, benzodiazepine, and serotonin-1A receptors.

β-Caryophyllene exerts anti-inflammatory effects by inhibiting primary inflammatory mediators, such as inducible nitric oxide synthase, interleukin-1β, Interleukin-6, tumor necrosis factor-alpha, nuclear factor kappa-light-chain-enhancer of activated B cells, cyclooxygenase 1, and cyclooxygenase 2 [42]. Additionally, β-caryophyllene enhances the characteristics of animals used as models for different inflammatory conditions, such as nervous system diseases, atherosclerosis, and cancer [42].

Essential oils extracted from leaves and stems inhibit NO production, reaffirming the pharmacological importance of *T. riparia*. Excessive NO production can cause the onset of several diseases and complications, including platelet inhibition, oxidative damage, cell death, complications related to diabetes, endothelial dysfunction, and neutrophil activation. These issues are linked to different signaling pathways and targets for pharmacological intervention [38].

Regarding the antiproliferative activity (Table 7), it was found that leaf CE, leaf EO, and stem CE exhibited notably superior antiproliferative activity against AGS cells, with GI_50_ values of 20, 34, and 40 µg mL^−1^, respectively. These values were 16.2, 27.64, and 32.5 times lower than the positive control, ellipticine (GI_50_ = 1.23 µg mL^−1^). Furthermore, leaf EO also demonstrated antiproliferative potential against CaCo-2 with a GI_50_ value of 30.89 µg mL^−1^, 25.52 times less efficient than ellipticine (GI_50_ = 1.21 ± 0.02 µg mL^−1^). It is worth noting that a direct comparison between the results of ellipticine and those achieved by EOs and CEs calls for caution, as ellipticine is a single compound and not a complex mixture, as are essential oils or crude extracts [43].

The antiproliferative activity of *T. riparia* has been investigated for some time by our research group, and in this sense, two diterpenes, 9β,13β-epoxy-7-abiethene and 6,7-dehydroroileanone, were isolated from *T. riparia* leaf essential oil [7]. The antitumor potential of the essential oil and isolated compounds was investigated by using the 3-(4,5-dimethylthiazol-2-yl)-2,5-diphenyl-2H-tetrazolium assay in different tumor cells. The essential oil and 9β,13β-epoxy-7-abietene showed high inhibitory power against human glioblastoma cancer cells (78.06% and 94.80%, respectively). The inhibition rates of the colon adenocarcinoma cell line were 85.00% and 86.54%, and for the human breast carcinoma cell line, these were 59.48% and 45.43%, respectively. The present study is the first to assess the antiproliferative potential of floral bud and twig EOs and CEs, suggesting that all parts of the plant should be examined, given the surprising results.

Regarding the antiproliferative potential of crude extracts, mainly CE from leaves and stems, phenolic diterpenes may have contributed to this activity. Cardoso et al. [44] attributed the antiproliferative effects to rosmarinidiphenol, rosmariquinone, and rosmanol. The authors reported that high concentrations of phenolic diterpenes interfere with the cell cycle, mostly during the interphase, by interrupting cytoplasmic replication and the onset of chromosome condensation. Overdoses of the diterpene carnosol affect dividing cells, acting on B1 cyclins during the process, making it impossible for the mitotic spindle to form properly.

Betulin, betulin aldehyde, and ketobetulinic acid were identified only in the CEs of leaves and stems (Table 1). Betulin and ketobetulinic acid are natural analogs of betulinic acid [3β-hydroxylup-20(29)-en-28-oic acid] and are lupine-type pentacyclic triterpenoids [45]. Their derivatives have a variety of pharmacological effects, such as anti-inflammatory, anti-HIV, antiparasitic, and antitumor activities. Studies have shown that betulinic acid induces programmed cell death by directly regulating the mitochondrial pathways and enhancing the generation of caspase-3, resulting in an antiangiogenic reaction. Betulin inhibits the TLR4/NF-κB pathway and reduces kidney, liver, and lung injuries in septic rats [46].

The SI of EOs ranged from 0.71 to 4.69; for CEs, the variation was from 1.26 to 8.8. The selectivity index assesses the correlation between the compound’s cytotoxicity for non-tumor cells and its effectiveness in tumor cells. A higher selectivity index indicates that these molecules have a stronger impact on the cells being tested, suggesting lower cytotoxicity. The results showed that the EOs and CEs presented a selectivity index greater than 1, except for the EOs of leaves and branches, which presented SIs of 0.71 and 0.87, respectively, for MCF-7 cells. According to Almeida et al. [47], an SI greater than 1 suggests higher activity against the target cell and lower activity against the other cells. Therefore, we can conclude that *T. riparia* crude extracts were not cytotoxic to the cells tested. Except for MCF-7 cells, the EOs did not show cytotoxicity against the cells tested.

### 3.3. Antiviral Activity

As far as we know, the antiviral activity of *T. riparia* is reported here for the first time. Our results show that the essential oils had a more potent antiviral effect than the extracts. In addition, extracts and essential oils from the leaves and stems were more active than those from the flowers. α-Pinene and β-pinene were among the compounds with higher concentrations in the EOs from stems and leaves. Both compounds showed antiviral activity against HSV-1 [48]. Specifically, α-pinene inhibits the binding and entry of the virus into the cell, with moderate inhibition of post-entry events [48]. In contrast, β-pinene inhibits only viral binding to cells [48]. As mentioned above, another compound found in higher concentrations in the EOs of leaves and stems was β-caryophyllene, which has been shown to exhibit antiviral activity against HSV-1 by preventing the virus from entering the cell [49]. Further research is needed to identify the active compounds responsible for *T. riparia*’s antiviral effects and understand their mechanisms of action.

According to the data, the *T. riparia* extract and EO from the leaves demonstrated the highest selectivity indexes, 15.01 and 12.67, respectively. This suggests that they may be valuable resources for developing novel antiviral products. This study is the initial evaluation of the chemical content, cellular antioxidant activity, inhibition of NO production, and antiproliferative and antiviral effects of essential oils and crude extracts from the flower buds and stems of *T. riparia*. More research is needed to explore their potential uses.

## 4. Materials and Methods

### 4.1. Plant Material and Botanical Identification

The culture of *T. riparia* was established in flowerbeds in the Medicinal Garden of Universidade Paranaense (UNIPAR) Umuarama, northwest region of Paraná State, Brazil, at coordinates S 23°46,225′ and WO 53°16,730′ and an altitude of 391 m. During the flowering period of *T. riparia*, which takes place in winter (June and July), leaves, floral buds, and stems were gathered.

Botanical analysis was conducted, and a specimen was placed in the Educational Herbarium of Universidade Paranaense, with reference number 2502. The species was documented in the National System of Genetic Heritage Management and Associated Traditional Knowledge (SisGen, as abbreviated in Portuguese) with identification number AD97496.

### 4.2. Collection and Extraction of Essential Oils and Preparation of Crude Extracts from Tetradenia riparia Leaves, Flower Buds, and Stems

*Tetradenia riparia* leaves, flower buds, and stems were dried at room temperature (35 °C), and the essential oils were extracted by hydrodistillation for 3 h by using a Clevenger-type apparatus [50]. Oil was collected and dried over anhydrous Na_2_SO_4_ and stored in amber glass flasks at −4 °C until use [50].

For extract preparation, leaves, floral buds, and stems were pulverized at 850 µm and subjected to a dynamic maceration process with ethanol–water (70%, *v v*^−1^). The plant powder and the extracting solution were kept in contact under gentle agitation at room temperature (23 ± 2 °C) until the plant material was utterly depleted. The extracts were then concentrated in a rotary evaporator (TE-210) at 40 °C to obtain crude extracts (CEs) [34].

### 4.3. Chemical Identification of Essential Oils

The chemical composition of the essential oils was determined by using gas chromatography–mass spectrometry (GC-MS). The analysis utilized an Agilent 7890B gas chromatograph paired with an Agilent 5977A mass spectrometer, equipped with an HP5-MS UI 5% capillary column (30 m × 250 µm × 0.25 µm; Agilent Technologies, Santa Clara, CA, USA) and an automatic injector (CTC PAL Control). To ensure effective separation of the analytes, 10 µL of essential oil was diluted in 1000 µL of dichloromethane, and 2 µL was injected into the column in split mode in a 1:30 ratio. The injector temperature was maintained at 260 °C, with helium as the carrier gas at a 1 mL min^−1^ flow rate. The oven temperature program began at 80 °C, followed by a ramp of 4 °C min^−1^ up to 260 °C, and a final ramp of 40 °C min^−1^ to 300 °C. The transfer line was set to 280 °C, while the ionization source and quadrupole temperatures were held at 230 °C and 150 °C, respectively. Mass spectrometric detection was conducted in electron ionization (EI) mode with a gain of 1.5, scanning within a mass-to-charge ratio (*m*/*z*) range of 40 to 550. A “solvent delay” of 3 min was implemented to avoid solvent interference. Identification of volatile compounds was achieved by comparing the obtained mass spectra with those in NIST Library (version 11.0) and by using retention indices (RIs) derived from a homologous series of n-alkane (C7-C40) standards [51].

### 4.4. Chemical Identification of Crude Extracts

For chemical characterization, a quantity of 1.0 mg from the crude extracts derived from *T. riparia* leaves, flower buds, and stems was solubilized in methanol (1 mL). The samples were subjected to analysis by using ultra-high-performance liquid chromatography (UHPLC; Nexera X2; Shimadzu, Kyoto, Japan) paired with a high-resolution mass spectrometer (qTOF Impact II; Bruker Daltonics Corporation, Billerica, MA, USA), which incorporated an electrospray ionization (ESI) source. The system operated in negative ionization mode with a capillary voltage of 4500 V and an end-plate potential set to −500 V. Dry gas conditions were maintained at a flow rate of 8 L min^−1^ and a temperature of 200 °C, while the nebulization gas pressure was set to 4 bar. Argon gas was employed with collision energies ranging between 15 and 30 eV for collision-induced dissociation. Data collection spanned the 50–1300 *m*/*z* range, acquiring 5 spectra per second. Automatic fragmentation of tandem mass spectrometry (MS/MS) data facilitated the selection of ions of interest. Chromatographic separation utilized a C18 column (75 × 2.0 mm i.d., 1.6 µm Shim-pack XR-ODS III, Kyoto, Japan), with a gradient elution composed of solvents A (water) and B (acetonitrile). The gradient program was as follows: starting with 5% solvent B from 0 to 1 min, increasing to 30% B from 1 to 4 min, then to 95% B from 4 to 8 min, and holding at 95% B from 8 to 17 min, at a column temperature of 40 °C. Compound identification followed protocols suggested in prior review studies on the genus *Tetradenia*, involving the calculation of mass error and comparison with data from MassBank (http://www.massbank.jp/) and Human Metabolome Database (http://www.hmdb.ca/).

### 4.5. Antioxidant Activity

#### 4.5.1. 2,2-Diphenyl-1-picrylhydrazyl (DPPH•) Method

The DPPH• assay was conducted based on the methodology outlined by Rufino et al. [52]. Essential oils and crude extracts were initially dissolved in methanol to achieve various concentrations (1.00, 0.75, 0.50, and 0.25 mg mL^−1^). A 10 μL aliquot of each diluted sample was mixed with 290 μL of a methanolic DPPH• solution (60 μM). For the negative control, 10 μL of the methanolic DPPH• solution (60 μM) was used. All mixtures were then incubated in the dark at room temperature for 30 min. After incubation, the absorbance was recorded at 515 nm by using a SpectraMax Plus 384 microplate reader (Molecular Devices, San Jose, CA, USA). The antioxidant capacity of the samples was quantified by using quercetin solution (60 μM) as a standard, representing 100% antioxidant activity. The absorbance-versus-concentration curve determined the concentration of the samples required to scavenge 50% of the free radicals (IC50).

#### 4.5.2. Ferric Reducing Antioxidant Power (FRAP)

The FRAP assay was carried out following the method initially established by Benzie and Strain [53] and subsequently modified by Rufino et al. [54] To prepare the FRAP reagent, 25 mL of acetate buffer (300 mM) was combined with 2.5 mL of 10 mM TPTZ solution and 2.5 mL of 20 mM ferric chloride solution. Crude extracts and essential oils were diluted in methanol to 1.00, 0.75, 0.50, and 0.25 mg mL^−1^. Next, 10 μL of these diluted samples and 290 μL of the FRAP reagent were pipetted into the wells of a 96-well microplate. The plate was then placed in a Spectra Max Plus 384 microplate reader (Molecular Devices, San Jose, CA, USA) vigorously shaken to ensure thorough mixing, and incubated at 37 °C for 30 min. Absorbance was recorded at 595 nm. Antioxidant activity was quantified by comparing the results against a standard curve prepared with ferrous sulfate (1000 μM).

#### 4.5.3. Quantification of Total Phenols

The total phenolic content of the samples was determined by the Folin–Ciocalteu method originally outlined by Swain and Hillis [55], with modifications suggested by Sousa de Sá et al. [56]. Both essential oils and crude extracts were diluted to 1.0 mg mL^−1^ in methanol. For the reaction, 155 μL of Folin–Ciocalteu reagent, 125 μL of sodium carbonate, and 20 μL of the diluted sample were combined in microplate wells. The mixture was then incubated in the dark for 60 min, and absorbance was recorded in triplicate by using a Spectra Max Plus 384 microplate reader (Molecular Devices, San Jose, CA, USA) at a wavelength of 760 nm. The calibration curve was generated by using seven concentrations of gallic acid (ranging from 0 to 100 μg mL^−1^) and was fitted with a linear regression model (Equation (1), R^2^ = 0.9997):

A = 0.0196C − 0.031(1)

where A is the sample absorbance and C is the gallic acid equivalent (GAE) concentration. The results were expressed as μg GAE mg^−1^ samples.

#### 4.5.4. β-Carotene–Linoleic Acid (BCLA) Method

The capacity of essential oils and crude extracts to inhibit the co-oxidation of β-carotene and linoleic acid was evaluated by following the procedure described by Rufino et al. [57]. Initially, 20 μL of linoleic acid, 265 μL of Tween 40, 25 μL of β-carotene solution (20 mg mL^−1^), and 0.5 mL of chloroform were combined in a beaker. The solvent was evaporated by using a dryer. Subsequently, the resultant emulsion was dissolved in 20 mL of hydrogen peroxide. To determine the antioxidant activity, 280 μL of this emulsion was mixed with 20 μL of the test samples diluted in methanol to achieve 1.00, 0.75, 0.50, and 0.25 mg mL^−1^ concentrations. The samples were incubated for 120 min, and their absorbance was recorded at 470 nm using a Spectra Max Plus 384 microplate reader (Molecular Devices, San Jose, CA, USA). A Trolox solution served as the control. The inhibition of oxidation was calculated by using Equations (2)–(4):

A_red_ = A_i_ − A_f_ (2)

O = [(A_red sample_ × 100]/(A_red system_) (3)

I = 100 − (O)(4)

where A_red_ is the reduction in the absorbance, A_i_ is the initial absorbance, A_f_ is the final absorbance, O is the oxidation percentage, and I is the inhibition percentage [58].

### 4.6. Cellular Antioxidant Activity

To assess cellular antioxidant activity (CAA), the procedure followed was that previously described by Fuente et al. [59]. Briefly, RAW 246.7 murine macrophages, commercially acquired from the European Collection of Authenticated Cell Cultures (ECACC), were routinely maintained with Dulbecco’s Modified Eagle Medium (DMEM) (HyClone, Logan, UT, USA) supplemented with L-glutamine (2 mM), penicillin (100 U mL^−1^), streptomycin (100 μg mL^−1^), fetal bovine serum (10%), and non-essential amino acids (2 mM) in T75 culture flasks at 37 °C in a humidified air incubator with 5% CO_2_ (Heal Force CO_2_ Incubator; Shanghai Lishen Scientific Equipment Co., Ltd., Shanghai, China).

The essential oils and crude extracts were dissolved in H_2_O:DMSO (50/50, *v v*^−1^) and in H_2_O, respectively, to a final concentration of 8 mg mL^−1^. This solution was then further successive diluted with 2′,7′-dichlorofluorescin (DCFH) prepared in ethanol and diluted with HBSS (50 μM) to obtain the final concentrations to be tested, ranging from 500 to 2000 μg mL^−1^.

Murine macrophages were detached by using a cell scraper, and after centrifugation, a solution with a cell density of 70,000 cells per mL was prepared. An aliquot (300 μL) was then transferred into black microplates with a clear bottom (SPL Life Sciences (Pocheon-si, Republic of Korea)), and the microplates were incubated. Once the cells reached confluence, the medium was discarded, and the cells were rinsed with HBSS (2×, 100 μL). They were then incubated for 1 h with the extracts at the different concentrations (200 μL; 500–2000 μg mL^−1^). After the incubation period, the cells were washed with HBSS (2×, 100 μL), and a solution of 2,2′-azobis(2-methylpropionamide) dihydrochloride (AAPH) (100 μL; 600 μM) was added. Fluorescence readings were taken every 5 min for 1 h by using a FLX800 microplate reader (Agilent, Santa Clara, CA, USA) at 485 nm excitation and 538 nm emission. Quercetin was used as the positive control, while DCFH solution and DMEM were tested as negative controls. The results were expressed as the percentage of inhibition at the highest concentration tested (2000 μg mL^−1^) [59].

### 4.7. NO Production Inhibition Assay

The ability of samples to inhibit nitric oxide (NO) production was assessed by using the method described by Corrêa et al. [60]. The essential oils and crude extracts were dissolved in H_2_O:DMSO (50:50, *v v*^−1^) and H_2_O, respectively, and successively diluted with water to obtain a range of concentrations to be tested (from 8 to 0.125 mg mL^−1^). The murine macrophage cell line (RAW 264.7) was routinely maintained under the conditions described in Section 4.6. The cells were detached by using a cell scraper, and a solution with 5 × 10⁵ cells mL^−1^ was prepared and transferred to 96-well plates. After 24 h of incubation, the cells were exposed to the studied samples at different concentrations and incubated for one hour. After this period, the cells were stimulated with lipopolysaccharide (LPS) (1 µg mL^−1^) (Sigma, St. Louis, MO, USA) for 24 h. Dexamethasone (50 µM) was tested as the positive control, while cells in the presence and absence of LPS were tested as negative controls.

The measurement of nitric oxide was carried out by using a Griess reagent kit (Promega Corporation, Madison, WI, USA). A standard curve for nitrite (sodium nitrite from 100 to 0.78 µM; y = 0.0068x + 0.0951; R^2^ = 0.9864) was prepared in a 96-well plate. After transferring 100 µL of the cell culture supernatant to a plate, the same volume of Griess reagent was added. The nitrite was quantified by measuring the absorbance at 540 nm (ELX800 microplate reader, Biotek Instruments, Winooski, VT, USA) and compared with the standard calibration curve. The obtained results were presented as EC_50_, representing the sample concentration responsible for 50% nitric oxide production inhibition in µg mL^−1^.

### 4.8. Antiproliferative Activity

The antiproliferative activity of the studied samples was assessed against four human tumor cells: gastric adenocarcinoma (AGS), colorectal adenocarcinoma (CaCo-2), breast adenocarcinoma (MCF-7), and lung carcinoma (NCI-H460). Additionally, the non-tumor cell line VERO (African green monkey kidney) was also tested. All the cell lines under investigation were regularly cultivated as adherent cell cultures in Gibco Roswell Park Memorial Institute (RPMI-1640) medium with the previously mentioned supplements (Section 4.6), except for the VERO cells, which were maintained in DMEM supplemented as described above. The studied samples were dissolved and successively diluted as described previously (Section 4.7). The concentrations tested were between 400 and 6.25 μg mL^−1^.

After detaching the cells with trypsinization, a solution at a density of 1.0 × 10^4^ cells per well was transferred to 96-well plates, except for VERO cells, which were seeded at 1.9 × 10^4^ cells per well. The sulforhodamine B (Extra synthesis, Genay, France) colorimetric assay was carried out by following the method previously described by Barros et al. [61]. Ellipticine was used as the positive control, and the cells without samples as the negative control. The results were expressed as the sample concentration responsible for inhibiting cell proliferation in 50% (GI_50_ values, µg mL^−1^).

The selectivity index (SI), which is the ratio between the cytotoxic concentration of 50% (GI_50_) for VERO cells and the GI_50_ for the tumor cells used, was also calculated with Equation (5).

SI = (GI_50_ of non-tumor cells)/(GI_50_ of tumor cells) (5)

### 4.9. Antiviral Activity

VERO cells were placed into 96-well plates at a density of 1 × 10^4^ cells per well in DMEM with 7.5% FBS. The next day, the cells were exposed to the virus at an MOI of 1.5 in DMEM with 2% FBS. After 1 h, the viral inoculum was removed, and DMEM with 2% FBS, with or without the natural product, was added. Each test was conducted in triplicate. Control-infected cells were exposed to DMSO during incubation. Uninfected control cells underwent the same treatment without adding the virus and were employed as a negative control. Finally, 48 h post-infection (h.p.i.), the supernatant was analyzed by using qPCR.

All qPCR reactions were carried out as described previously [62]. In brief, the viral genome was quantified by utilizing 5 µL of (Bio-Rad, Hercules, CA, USA), 500 pM of ICP0 FW (5′-GTCGCCTTACGTGAACAAGAC-3′), ICP0 RV (5′-GTCGCCATGTTTCCCGTCTG-3′), and 1 µL of the sample. The amplifications were conducted by using a StepOnePlus™ thermocycler (Thermo Fischer Scientific, Waltham, MA, USA). The two-step program comprised initial denaturation for 5 min at 95 °C, 35 cycles of 15 s at 95 °C, and 30 s at 60 °C, followed by a final melting curve. Viral genome copies (VGCs) were calculated by utilizing a calibration curve. Reactions without templates were used as negative controls.

### 4.10. Statistical Analysis

The results were expressed as arithmetic average values ± standard deviation. The data underwent analysis of variance (ANOVA) and were then compared by using Tukey’s test (*p* ≤ 0.05) with the SPSS Statistics 22 software. StatSoft Statistics 10.0, South America, 2022, was utilized for the analysis.

## 5. Conclusions

The presence of anthocyanins, phenolic acids, flavonoids, tannins, and terpenes in *Tetradenia riparia* leaf, stem, and floral bud crude extracts (CEs) and of sesquiterpenes and diterpenes in essential oils (EOs) reinforce the antioxidant potential of this species. The crude extract from the leaves exhibited the highest level of inhibition of oxidation (102.25%) in the β-carotene–linoleic acid co-oxidation system and cellular antioxidant activity (82%). The crude extract from the stems demonstrated a significant regenerative effect on the DPPH radical, with an IC_50_ of 0.51 ± 0.03 mL^−1^, and the highest total phenol content, 111.68 µg of gallic acid mg^−1^ of sample.

When evaluated in tumor cells, the leaf CE and EO and the stem CE showed better results against AGS cells, and the EO from the leaves also showed potential against Caco-2. The stem EO demonstrated anti-inflammatory potential (EC_50_ = 76 µg mL^−1^).

In terms of antiviral activity, the leaf EO demonstrated the highest activity (EC_50_ = 9.64 µg mL^−1^, SI: 15), while the flower bud CE displayed the lowest activity (EC_50_ = 24.55 µg mL^−1^, SI: 7.49).

## Figures and Tables

**Table 1:** Analysis of chemical composition of crude extracts from leaves, stems, and flower buds of *Tetradenia riparia* using UHPLC-ESI-qTOF-MS/MS.

Compound	Molecular Formula	Theoretical *m*/*z* [M − H]^−^	Experimental *m*/*z* [M − H]^−^	Error (ppm)	Rt (min)	Sample
Anthocyanins						
Pelargonidin	C_15_H_11_O_5_^+^	271.0601 [M]^+^	271.0594	2.58	4.41	Leaves
			271.0593	2.95	4.43	Stems
			271.0592	3.32	4.42	Flower buds
Cyanidin	C_15_H_11_O_6_^+^	287.0550 [M]^+^	287.0542	2.79	4.27	Leaves
			287.0543	2.44	4.25	Stems
			287.0541	3.13	4.25	Flower buds
Malvidin	C_17_H_15_O_7_^+^	331.0812 [M]^+^	331.0804	2.42	4.48	Leaves
			331.0803	2.72	4.46	Stems
			331.0803	2.72	4.46	Flower buds
Flavonoids						
Astragalin	C_21_H_20_O_11_	447.0921 [M − H]^−^	447.0903	4.03	4.21	Leaves
			447.0906	3.36	4.44	Stems
			447.0904	3.80	4.17	Flower buds
Luteolin	C_15_H_10_O_6_	285.0393 [M − H]^−^	285.0389	1.40	5.16	Leaves
			285.0388	1.75	5.14	Stems
			285.0387	2.10	5.15	Flower buds
Apigenin	C_15_H_10_O_5_	269.0444 [M − H]^−^	269.0439	1.86	5.40	Leaves
			269.0439	1.86	5.44	Stems
			269.0438	2.23	5.42	Flower buds
Phenolic acids						
Sagerinic acid	C_36_H_32_O_16_	721.1763 [M + H]^+^	721.1734	4.02	4.12	Leaves
			721.1732	4.29	4.10	Flower buds
4-Hydroxybenzoic acid	C_7_H_6_O_3_	137.0233 [M − H]^−^	137.0233	0	4.03	Leaves
			137.0233	0	3.99	Stems
			137.0234	−0.73	4.01	Flower buds
*p*-Coumaric acid	C_9_H_8_O_3_	163.0389 [M − H]^−^	163.0389	0	4.58	Leaves
			163.0389	0	4.59	Stems
			163.0388	0.61	4.60	Flower buds
Ferulic acid	C_10_H_10_O_4_	193.0495 [M − H]^−^	193.0492	1.55	4.72	Leaves
			193.0495	0	4.74	Stems
			193.0489	3.11	4.75	Flower buds
Protocatechuic acid	C_7_H_6_O_4_	153.0182 [M − H]^−^	153.0182	0	4.78	Leaves
Caffeic acid	C_9_H_8_O_4_	179.0350 [M − H]^−^	179.0336	7.82	4.29	Leaves
			179.0336	7.82	4.25	Stems
			179.0335	8.38	4.19	Flower buds
Rosmarinic acid	C_18_H_16_O_8_	361.0917 [M + H]^+^	361.0905	3.32	4.11	Leaves
			361.0903	3.88	4.10	Flower buds
Tannins						
Procyanidin	C_30_H_26_O_13_	593.1289 [M − H]^−^	593.1266	3.88	5.46	Leaves
Terpenes						
Sandaracopimaradienolal	C_20_H_30_O_2_	303.2318 [M + H]^+^	303.2307	3.63	4.52	Leaves
			303.2310	2.64	4.50	Stems
			303.2306	3.96	4.87	Flower buds
14-Hydroxyhumulene	C_15_H_24_O	221.1899 [M + H]^+^	221.1894	2.26	5.31	Leaves
			221.1892	3.16	5.27	Stems
			221.1892	3.16	5.27	Flower buds
Abieta-7,9 (11) -dien-13β-ol	C_20_H_32_O	289.2525 [M + H]^+^	289.2517	2.77	8.82	Leaves
			289.2511	4.84	8.82	Flower buds
8(14),15-Sandaracopimaradiene-2α, 18-diol	C_20_H_32_O_2_	305.2475 [M + H]^+^	305.2463	3.93	6.55	Leaves
			305.2464	3.60	6.17	Stems
			305.2463	3.93	6.18	Flower buds
Campesterol	C_28_H_48_O	423.3597 [M + H]^+^	423.3601	−0.94	13.87	Leaves
			423.3600	−0.71	13.84	Flower buds
Eugenol	C_10_H_12_O_2_	165.0910 [M + H]^+^	165.0906	2.42	6.14	Stems
Carnosic acid	C_20_H_28_O_4_	333.2060 [M + H]^+^	333.2053	2.10	7.02	Leaves
			333.2051	2.70	6.51	Stems
			333.2044	4.80	6.99	Flower buds
Carnosol	C_20_H_26_O_4_	331.1903 [M + H]^+^	331.1892	3.32	6.94	Leaves
			331.1893	3.02	6.94	Stems
			331.1892	3.32	6.94	Flower buds
Rosmanol	C_20_H_26_O_5_	347.1853 [M + H]^+^	347.1843	2.88	6.62	Leaves
			347.1840	3.74	6.63	Flower buds
Rosmarinidifenol	C_20_H_28_O_3_	317.2111 [M + H]^+^	317.2100	3.47	6.13	Leaves
			317.2105	1.89	6.34	Stems
			317.2099	3.78	6.01	Flower buds
Rosmariniquinone	C_19_H_22_O_2_	283.1692 [M + H]^+^	283.1685	2.47	8.30	Stems
Betulin aldehyde	C_30_H_48_O_2_	441.3727 [M + H]^+^	441.3706	4.76	10.04	Leaves
Betulin	C_30_H_50_O_2_	443.3883 [M + H]^+^	443.3869	3.16	11.45	Leaves
			443.3862	4.74	11.50	Stems
Ketobetulinic acid	C_30_H_46_O_4_	471.3468 [M + H]^+^	471.3451	3.61	5.21	Leaves
			471.3455	2.76	5.25	Stems
			471.3454	2.97	5.22	Flower buds
Maslinic acid	C_30_H_48_O_4_	473.3625 [M + H]^+^	473.3609	3.38	6.57	Leaves
			473.3615	2.11	6.59	Stems
			473.3611	2.96	5.57	Flower buds
Sandaracopimaradienediol	C_20_H_32_O_2_	305.2475 [M + H]^+^	305.2464	3.60	5.46	Leaves
			305.2464	3.60	6.17	Stems
			305.2462	4.26	6.17	Flower buds
Sandaracopimaric acid	C_20_H_30_O_2_	303.2318 [M + H]^+^	303.2312	1.98	5.68	Leaves
			303.2309	2.97	5.70	Stems
			303.2306	3.96	6.41	Flower buds
6,7-Dehydroroyleanone	C_20_H_26_O_3_	315.1954 [M + H]^+^	315.1930	7.61	5.67	Leaves
			315.1943	3.49	5.77	Stems
			315.1943	3.49	6.12	Flower buds

Rt: retention time.

**Table 2:** Chemical composition of the essential oils derived from the leaves, flower buds, and stems of *Tetradenia riparia*.

Peak	RT	Compounds	RI Lit.	RI Calc.	Relative Area (%)			Identification Methods
					Leaves	Flower Buds	Stems	
1	3.178	α-Pinene	932	930	1.6	0.5	1.8	a. b. c
2	3.369	Camphene	946	935	1.2	0.4	1.6	a. b. c
3	3.645	Sabinene	969	975	1.4	0.5		a. b. c
4	3.722	β-Pinene	974	986	0.7	0.3	1.0	a. b. c
5	4.463	Limonene	1024	1030	1.2	0.5	0.3	a. b. c
6	4.533	Trans-β-ocimene	1044	1041	0.5	0.3	-	a. b. c
7	5.624	Fenchone	1083	1094	11.6	4.7	0.4	a. b. c
8	6.106	Endo fenchol	1114	1119	0.8	0.4	-	a. b. c
9	6.834	Camphor	1141	1156	2.4	0.9	0.1	a. b. c
10	7.317	Endo-carvacrol	1165	1178	0.8	0.4	1.5	a. b. c
11	7.593	Terpinen-4-ol	1174	1190	0.5	-	-	a. b. c
12	7.912	α-Terpineol	1186	1196	0.4	0.3		a. b. c
13	11.930	δ-Elemene	1335	1332	0.3	0.3	0.4	a. b. c
14	13.067	α-copaene	1374	1357	0.9	0.8	0.3	a. b. c
15	13.523	β-Elemene	1389	1377	0.7	0.5	-	a. b. c
16	14.059	α-Gurjunene	1409	1403	1.7	1.5	1.2	a. b. c
17	14.371	β-Caryophyllene	1417	1416	5.7	3.9	5.0	a. b. c
18	14.774	α-Bergamotene	1432	1430	0.8	0.6	0.4	a. b. c
19	14.922	Humulene	1436	1432	0.6	0.5	2.5	a. b. c
20	15.317	Aromadendrene	1439	1438	0.4	0.3	1.2	a. b. c
21	15.476	Allo-aromadendrene	1458	1459	-	-	1.9	a. b. c
22	15.560	Aromadendrene dehydro-	1460	1461	-	-	2.4	a. b. c
23	15.971	γ-Muurolene	1478	1475	0.3	0.2	0.3	a. b. c
24	16.205	Germacrene D	1484	1489	-	-	1.0	a. b. c
25	16.206	β-Guaiene	1492	1490		0.2	0.5	a. b. c
26	16.342	Valenceno	1496	1493	0.3	-	-	a. b. c
27	16.433	Viridiflorene	1496	1495	0.6	0.6	-	a. b. c
28	16.658	α-Muurolene	1500	1500	0.8	0.6	-	a. b. c
29	16.612	β-Himachalene	1500	1502	0.5	1.6	0.3	a. b. c
30	16.658	Byciclogermacrene	1500	1505	3.7	3.2	6.0	a. b. c
31	16.856	α-Farnesene	1505	1512	1.2	0.3	-	a. b. c
32	17.061	γ-Cadinene	1513	1514	1.2	0.9	1.4	a. b. c
33	17.230	7-Epi-α-selinene	1520	1519	1.3	2.0	1.1	a. b. c
34	17.233	δ-Cadinene	1523	1520	4.6	3.3	2.7	a. b. c
35	17.239	γ-Dehydro-arhimachalene	1532	1524	0.8	-	0.5	a. b. c
36	17.345	10-Epi-cubebol	1533	1530	0.2	1.3	0.4	a. b. c
37	17.409	Palustrol	1567	1564	-	0.2	0.4	a. b. c
38	17.720	Germacrene D-4-ol	1574	1573	-	-	0.9	a. b. c
39	17.745	Spathulenol	1577	1579	2.9	-	9.1	a. b. c
40	17.896	Caryophyllene oxide	1582	1587	1.3	4	4.6	a. b. c
41	18.049	Globulol	1590	1592	-	1.0	0.5	a. b. c
42	18.054	Viridiflorol	1592	1592	0.3	0.3	0.2	a. b. c
43	18.351	Carotol	1594	1600	-	-	1.1	a. b. c
44	18.464	Ledol	1602	1605	0.5	0.5	1.3	a. b. c
45	18.581	α-Acorenol	1632	1629	1.6	0.2	0.1	a. b. c
46	18.817	Epi-α-cadinol	1638	1636	3.4	2.8	0.8	a. b. c
47	19.295	Allo-aromadendrene epoxide	1639	1637	-	-	0.6	a. b. c
48	19.921	Epi-α-muurolol	1640	1642	0.3	0.3	0.6	a. b. c
49	20.207	Caryophyll-4(12),8(13)-dien	1640	1645	-	-	0.4	a. b. c
50	20.274	α-Muurolol	1644	1647	3.4	3.4	0.6	a. b. c
51	20.413	Cubenol	1646	1649	0.2	-	0.3	a. b. c
52	20.528	β-Eudesmol	1649	1650	-	1.6	0.1	a. b. c
53	20.683	α-Cadinol	1652	1657	12.2	10.7	7.4	a. b. c
54	20.771	14-Hydroxy-9-epi-caryophyllene	1668	1671	8.6	14.2	5.1	a. b. c
55	20.931	2,3-Dihydrofarnesol	1688	1696	-	-	0.9	a. b. c
56	21.047	Siobinol	1688	1700	-	-	3.2	a. b. c
57	21.378	Cedrenol	-	1702	-	-	2.1	a. b. c
58	21.470	Guaiol acetate	1725	1731	-	-	1.5	a. b. c
59	22.056	Valerenol	-	1733	-	-	2.2	a. b. c
60	22.381	γ-Costol	1746	1745	-	-	4.1	a. b. c
61	22.459	Cis-lanceol	1760	1770	-	-	2.2	a. b. c
62	28.070	Cembrene	1937	1920	-	1.0	-	a. b. c
63	29.476	9β,13β-Epoxi-7-abietene	-	1945	7.3	8.8	4.3	d*
64	29.537	Manooil oxide	1987	1957	0.3	0.5	0.1	a. b. c
65	31.389	Abieta-8,11,13-triene	2055	2051	1.4	1.5	0.2	a. b. c
66	31.595	Abietadiene	2087	2085	0.3	5.5	0.2	a. b. c
67	33.409	n.i.	-	2087	-	1.0	-	a. b. c
68	33.859	6,7-Dehydroroyleanone	-	2094	5.8	7.5	7.5	a. b. c
69	37.330	n. i	-	2105	-	1.6	-	a. b. c
		Total Identified			99.6	96.7	98.3	
		Monoterpene hydrocarbons			6.6	2.3	4.3	
		Oxygenated monoterpenes			16.4	6.8	2.0	
		Sesquiterpene hydrocarbons			26.4	21.5	29.3	
		Oxygenated sesquiterpenes			35.0	41.2	50.5	
		Diterpenes			15.1	24.8	12.3	
		Unidentified			-	2.6	-	

a: Substances listed based on their elution sequence in the HP-5 MS column. b: RI = retention index (RI) determined by using n-alkanes C7 to C40 in an HP-5 MS column. c: Identification was established by comparing it with the mass spectrum from NIST 11.0 Libraries. Relative area (%): percentage of the chromatogram area occupied by the substances. n.i. = unidentified. RT = retention time. (-): substance not present. d*: Recognition using Nuclear Magnetic Resonance (NMR) [7].

**Table 3:** Inhibition potential of β-carotene–linoleic acid co-oxidation of essential oils and crude extracts obtained from leaves, flower buds, and stems of *Tetradenia riparia*.

Samples	Plant Parts	Concentrations (mg mL^−1^)			
		1.00	0.75	0.50	0.25
Essential oils	Leaves	78.99 ± 0.27 ^Ab^	76.98 ± 0.08 ^Bc^	68.00 ± 0.08 ^Cd^	67.45 ± 0.16 ^Dd^
	Stems	76.13 ± 5.20 ^Ab^	76.75 ± 0.25 ^Ac^	73.72 ± 0.20 ^Ac^	70.76 ± 0.07 ^Ac^
	Flower buds	80.28 ± 0.20 ^Ab^	78.08 ± 0.2 ^Bb^	75.14 ± 0.16 ^Cb^	74.41 ± 0.16 ^Db^
Crude extracts	Leaves	102.25 ± 0.07 ^Aa^	99.54 ± 0.3 ^Ba^	98.55 ± 0.20 ^Ca^	95.98 ± 0.32 ^Da^
	Stems	65.94 ± 0.07 ^Ac^	61.41 ± 0.20 ^Be^	56.27 ± 0.12 ^Cf^	55.07 ± 0.30 ^Df^
	Flower buds	69.46 ± 0.16 ^Ac^	64.58 ± 0.3 ^Bd^	61.50 ± 0.23 ^Ce^	57.86 ± 0.19 ^De^

The results represent the means ± standard deviation of three separate assays. The data underwent analysis of variance (ANOVA), and distinctions between averages were evaluated by using Tukey’s test (*p* ≤ 0.05). Values in the same column and marked with different lowercase letters and values in the same row indicated by different uppercase letters demonstrate significant differences (*p* ≤ 0.05). Trolox (0.2 mg mL^−1^) served as the positive control. CE refers to crude extract, while EO refers to essential oil.

**Table 4:** Antioxidant activity determined by 2,2-diphenyl-1-picrylhydrazyl radical scavenging and ferric reducing antioxidant power assays, and total phenolic content of essential oils and crude extracts obtained from leaves, flower buds, and stems of *Tetradenia riparia*.

Samples	Plant Parts	DPPH	FRAP	Total Phenols
		IC_50_ (mg mL^−1^)	(µM Ferrous Sulfate mg^−1^)	(µg GAE mg^−1^)
Essential oils	Leaves	5.62 ± 0.69 ^d^	0.44 ± 0.00 ^d^	10.70 ± 0.55 ^f^
	Stems	8.47 ± 0.50 ^e^	0.44 ± 0.00 ^d^	15.37 ± 0.74 ^e^
	Flower buds	4.47 ± 0.60 ^c^	0.45 ± 0.00 ^d^	27.07 ± 0.72 ^d^
CrudeMILOSextracts	Leaves	1.91 ± 0.01 ^b^	0.35 ± 0.03 ^e^	75.60 ± 0.36 ^b^
	Stems	0.51 ± 0.03 ^a^	0.50 ± 0.01 ^c^	111.68 ± 0.66 ^a^
	Flower buds	0.91 ± 0.05 ^ab^	0.81 ± 0.01 ^b^	65.22 ± 0.60 ^c^
Quercetin	-	0.01 ± 0.01 ^a^	-	-
Trolox	-	-	9.17 ± 0.01 ^a^	-

The results represent the means ± standard deviation of three separate assays. The data underwent analysis of variance (ANOVA), and distinctions between averages were evaluated by using Tukey’s test (*p* ≤ 0.05). Values sharing the same column and marked with different letters demonstrate significant differences (*p* ≤ 0.05). Quercetin (0.0103 mg mL^−1^) and Trolox (9.175 mg mL^−1^) were positive controls in the DPPH• and FRAP assays. IC_50_ = half-maximal inhibitory concentration; DPPH• = 2,2-diphenyl-1-picrylhydrazyl; FRAP = ferric reducing antioxidant power; GAE = gallic acid equivalent; CE refers to crude extract, while EO refers to essential oil.

**Table 5:** Cellular antioxidant activity (CAA) of *Tetradenia riparia* flower buds, leaves, and stem essential oil and crude extract.

Samples	Plant Parts	Inhibition at Maximum Concentration Tested (%)
Essential oils	Leaves	64%
	Stems	45%
	Flower buds	46%
Crude extracts	Leaves	82%
	Stems	47%
	Flower buds	54%

Quercetin (% inhibition at 0.3 μg mL^−1^): 95 ± 5.

**Table 6:** *Tetradenia riparia* flower buds, leaves, and stems essential oils’ and crude extracts’ NO production inhibition in murine macrophages (RAW 264.7) stimulated by LPS.

	NO Production Inhibition		
	Samples	Plant Parts	EC_50_ (µg mL^−1^)
Murine macrophage cells	Essential oils	Leaves	95 ± 1 ^c^
		Stems	76 ± 1 ^b^
		Flower buds	247 ± 5 ^f^
	Crude extracts	Leaves	192 ± 11 ^de^
		Stems	185 ± 5 ^d^
		Flower buds	205 ± 2 ^dc^
	Dexamethasone	-	16 ± 1 ^a^

The results represent the means ± standard deviation of three separate assays. Values sharing the same column and marked with different letters demonstrate significant differences according to Tukey’s HSD test (*p* ≤ 0.05). EC_50_ = concentration that is efficient in reducing the production of nitric oxide by 50%.

**Table 7:** Antiproliferative activity (GI_50_ µg mL^−1^) and selectivity index (SI) of essential oils and crude extracts obtained from flower buds, leaves, and stems of *Tetradenia riparia*.

Samples	Plant Parts	AGS	SI	CaCo-2	SI	MCF-7	SI	NCI-H460	SI	VERO
Essential oils	Leaves	34 ± 3 ^cA^	4.26	31 ± 2 ^bA^	4.69	204 ± 17 ^dC^	0.71	134 ± 7 ^dB^	1.08	145 ± 9 ^bB^
	Stems	50.0 ± 0.4 ^dA^	2.86	41 ± 4 ^bcA^	3.48	163 ± 14 ^cB^	0.87	55 ± 4 ^bA^	2.6	143 ± 11 ^bB^
	Flower buds	42 ± 4 ^cdA^	4.30	63 ± 5 ^dA^	2.87	174 ± 17 ^cdB^	1.04	149 ± 14 ^dB^	1.21	181 ± 16 ^cB^
CrudeMILOSextracts	Leaves	20 ± 1 ^bA^	8.8	52 ± 4 ^cdB^	3.38	59 ± 4 ^bB^	2.98	77 ± 2 ^cC^	2.28	176 ± 3 ^bcD^
	Stems	40 ± 3 ^cA^	4.75	107 ± 7 ^fC^	1.77	71 ± 7 ^dB^	2.67	75 ± 1 ^cB^	2.53	190 ± 15 ^cD^
	Flower buds	67 ± 7 ^eA^	2.74	83 ± 3 ^eA^	2.21	145 ± 12 ^cB^	1.26	74 ± 6 ^cA^	2.48	184 ± 18 ^cC^
Ellipticine		1.23 ± 0.03 ^aB^	1	1.21 ± 0.02 ^aB^	1	1.02 ± 0.02 ^aA^	1	1.01 ± 0.01 ^aA^	0.91	1.4 ± 0.1 ^aC^

The results represent the means ± standard deviation of three separate assays. Values sharing the same column and marked with different lowercase letters, as well as values within the same row indicated by different uppercase letters, demonstrate significant differences according to Tukey’s HSD test (*p* ≤ 0.05). GI_50_ = concentration that reduces the growth by 50%; SI = selectivity index. Ellipticine served as the positive control. AGS (gastric adenocarcinoma). CaCo-2 (colorectal adenocarcinoma). MCF-7 (breast adenocarcinoma). NCI-H460 (lung carcinoma). African green monkey kidney epithelial cells (VERO).

**Table 8:** Antiviral activity of essential oils and crude extracts of *Tetradenia riparia*.

Samples	Plant Parts	IC_50_ (µg mL^−1^)	SI
Essential oils	Leaves	9.64	15.01
	Stems	11.75	12.17
	Flower buds	24.24	7.46
Crude extracts	Leaves	18.84	9.34
	Stems	15.01	12.65
	Flower buds	24.55	7.49

## Data Availability

The datasets generated during and/or analyzed during the current study are available from the corresponding author upon reasonable request.
